# Bioethanol from wheat straw hydrolysate solubility and stability in waste cooking oil biodiesel/diesel and gasoline fuel at different blends ratio

**DOI:** 10.1186/s13068-023-02264-9

**Published:** 2023-02-01

**Authors:** Mostafa M. El-Sheekh, Aya A. El-Nagar, Medhat ElKelawy, Hagar Alm-Eldin Bastawissi

**Affiliations:** 1 Botany Department, Faculty of Science, Tanta University, Tanta, 31527 Egypt; 2 Microbial Biotechnology Department, Genetic Engineering and Biotechnology Research Institute, Sadat City University, El Sadat City, Egypt; 3 Mechanical Power Engineering Departments, Faculty of Engineering, Tanta University, Tanta, Egypt

**Keywords:** Biofuel solubility, Emulsion, Blends stability, Ignition delay, Emission, Performance, Combustion, Biofuel particle size

## Abstract

The work focuses on studying the solubility and stability of dissolved bioethanol as a fuel additive in different fuel blends of gasoline, diesel, 50% diesel/50% biodiesel. Dissolved ethanol fuel appears as particles with a unique size distribution inside the whole fuel blends, and its stability was measured in this work. Bioethanol dissolved fuel particles stability was improved after blending the bioethanol with 50% diesel/50% biodiesel than pure diesel or pure gasoline fuel alone. The obtained results reveal that the lowest bioethanol particles stability was obtained when commixed with gasoline and the suspended ethanol particles completely accumulated at different concentrations of bioethanol in the fuel blends of 2%, 4%, 6%, 8%, 10%, and 12% by volume after 1 h of mixing time. Furthermore, the measured data of the bioethanol particles size distribution reveals that the suspended stability in the diesel blend improve slightly for all bioethanol concentrations of 10%, 15%, 20%, 25%, and 30% by volume. While the bioethanol concentrations of 5% show acceptable particles stability and size distribution during the whole experiments time. Obtained results show that bioethanol suspended particles stability was enhanced for 50% diesel/50% biodiesel blend with different bioethanol concentrations of 5%, 10%, 15%, 20%, 25%, and 30% by volume basis. However, the size of the particles increased as the bioethanol concentration rose with the passage of time.

## Introduction

During the last period time, the majority of the researchers for the new resources of renewable fuels were seeking to enhance the combustion behaviors and reduce the pollutant emission by substituting lower alcohols with conventional fuels [1]. The proposed technique of replacing the lower alcohols was considered a new solution because of the expected advantages gained, such as the economical and technological issues [2]. The replacing the lower alcohols with diesel and diesel/biodiesel blended fuel has attracted not a few researchers to improve internal combustion engine efficiency [3]. However, the obtained results reveal that by increasing the n-pentane as a lower alcohol additive with different volume percentages in the diesel/biodiesel fuel blends, the efficiency has improved significantly.

Air pollution and the threat of fossil fuel depletion have increased interest in seeking ecologically friendly and sustainable alternative energy sources. The production of biodiesel from an oil mixture of soybean and sunflower has been optimized and utilized as a new alternative fuel resource in the diesel engine. The results show a dramatic enhancement in engine thermal efficiency and the tailpipe exhausts. As a renewable energy source, bioethanol is one of the most widely used alternative fuels in many countries to reduce total dependency on fossil fuels [4]. Bioethanol has a key role in reducing crude oil use. Ethanol's high oxygen content helps to improve fuel blend combustion, resulting in lower hazardous exhaust emissions compared to gasoline and diesel, supporting a cleaner environment in the future [5].

Also, the effect of adding 10% and 20% by volume of bioethanol as an lower alcohol additives to a mixture of WCO biodiesel/diesel#1 on internal combustion engine performance and emissions have been investigated [6]. The obtained results elucidated that the engine NOx has reduced, while the engine brake power is dramatically increased. The authors claimed that the bioethanol additives have a pivotal role in enhancing the spray and fuel droplets distribution inside the engine combustion chamber [7]. The main objective of adding lower alcohol such as ethanol was to enhance the phenomenon known as the “micro-explosion,” which was responsible for increasing the fuel spray behaviors during the combustion process. However, ethanol (C_2_H_3_OH) is the most famous liquid and lower alcohol fuel additives which has OH radicals and two carbon molecules with a relatively high boiling point. Methanol has been financially used to extend the combustion phasing because of its higher heating power and its capacity to combust with low smoke emissions. It has specific properties, for example, higher ignition delay and more modest cetane number, which jumbles the immediate double-dealing of methanol in CI motors [8]. The ethanol and gasoline fuel blends with different percentages have been investigated to elucidate the phase stability [9]. In this work, different ethanol blends of 5%, 10%, 15%, and 20% havebeen applied. The results elucidated that the phase stability of ethanol in the mixture can enhance using Cyclohexanol as a stabilizing agent.

Bioethanol is typically made by fermenting sugars derived from molasses and fruits. It is also made from starchy ingredients, including grains, corn, and wheat. Currently, lignocellulosic biomass and other sucrose-containing materials are utilized as cost-effective ethanol feedstocks [10]. There are varieties of reasons why virtually dry ethanol must be blended into gasoline. Phase separation is one of the most important issues that might arise in the formulation of ethanol fuel, especially in cold climates with high ambient humidity. The hygroscopicity of ethanol can cause a phase separation [11]. Because of the separated corrosive mixture of ethanol and water, this problem can result in a decline in the quality of the fuel formulation, as well as corrosion of engine parts and storage tanks [12]. Some co-solvents and surfactants were explored for stabilizing hydrous ethanol–gasoline formulations and demonstrated a substantial efficiency in the phase stability of these blends [11]. The stability of the emulsion is significantly influenced by the concentration of the surfactants [13]. The amount of ethanol can also alter how stable an emulsion is. Low ethanol concentrations can improve emulsion stability by reducing oil droplet size as a result of a lower interfacial tension [14, 15]. Nevertheless, excessive ethanol content alters the solubility of surfactants and results in emulsion instability [13, 16] found that raising the emulsifier concentration to up to 10% of the total volume decreases the emulsion’s droplet size and improves the stability of the emulsified gasoline. In the literature, emulsifiers or co-solvents are necessary to have been studied to stabilize bioethanol inside fuel blends [17]. Biodiesel (FAME) has received a lot of attention among possible co-solvents because of its full miscibility with both diesel fuel and ethanol, as well as its renewable nature [18].

Due to its excellent thermal efficiency and low emissions, the dual or multi-fuel components combustion mode has received a great deal of attention. Form this point; the blended fuel homogeny and its satiability have a major effect on the combustion efficiency. In conclusion, this work demonstrates that a key element in enhancing combustion qualities is the stability and solubility of bioethanol in “gasoline, diesel, 50% diesel/50% biodiesel” blends. This examination is based on optical and microscopic observation of droplet size distribution. After combining bioethanol with 50% diesel and 50% biodiesel, the fuel particles remain stable.

## Novelty of the present study

The main problem of using multifuel components in an internal combustion engine is the homogeneity and stability of the blended fuel. As stated in the above introduction, biodiesel fuel has been used as a new renewable energy source instead of gasoil fuel with different portions. However, according to the author’s knowledge, there is no available data in the literature concerning the distribution of the blended fuel in the main gasoil fuel. In this context, the novelty of the present study is investigating the distribution of dissolved fuel additives such as bioethanol and their stability with time. Therefore, the stability and solubility of bioethanol inside fuel blends is an important new technology for studying the particles distribution of dissolved bioethanol also statistical analysis for these particles. This work represents a contribution to the scientific literature by improving bioethanol particles stability and their distribution through three tested blends: “gasoline, diesel, 50% diesel/50% biodiesel”. The solubility and stability of dissolved bioethanol as a fuel additive in different fuel blends of gasoline, diesel, 50% diesel/50% biodiesel will be investigated experimentally. Dissolved ethanol fuel particles size distribution inside the whole fuel blends and their stability were measured in this work using microscopic photos. Bioethanol dissolved fuel particles stability after blending the bioethanol with 50% diesel/50% biodiesel, diesel, and pure gasoline fuel alone will be studied.

## Materials and methods

### Materials

Bioethanol employed in this study was obtained from the fermentation of wheat straw hydrolysate using Aspergillus niger strain MZ062603 [19]. Biodiesel was produced from restaurant waste cooking oil [6, 20, 21]. The elucidated results of the transesterification process have been optimized by investigating the effect of biodiesel production parameters such as methanol to oil ratio, NaOH concentration, reaction time, and all other parameters on biodiesel yield. In addition, the effect of using biodiesel with different blends dose with gasoil on the diesel engine performance and emissions characteristics have been applied [22, 23].

### Fuel preparation

In the present study, the three fuel blends have been prepared. The gasoline fuel, diesel fuel, and 50% diesel/50% biodiesel fuel blends were added to different concentrations of produced bioethanol as follows: gasoline blend was prepared by mixing the 2%, 4%, 6%, 8%, 10%, and 12% *v*/*v* of bioethanol well with gasoline. In addition,the diesel blend was prepared by mixing well 5%, 10%, 15%, 20%, 25%, and 30% *v*/*v* of produced bioethanol with diesel. Finally, 50%diesel/50% biodiesel blend was prepared by blending well the 5%, 10%, 15%, 20%, 25%, and 30% v/v of obtained bioethanol with 50%diesel/50% biodiesel. The two-dimensional surface model has used to evaluate the blended fuel density as mentioned by Mert Gülüm and Atilla Bilgin [24, 25]. The obtained results demonstrated the values of the density of different fuel blend ratio at different temperatures rages of 278.15–368.15 K at the standards condition.

### Emulsion stability evaluation

#### Visual investigation

**Fig. 1:**
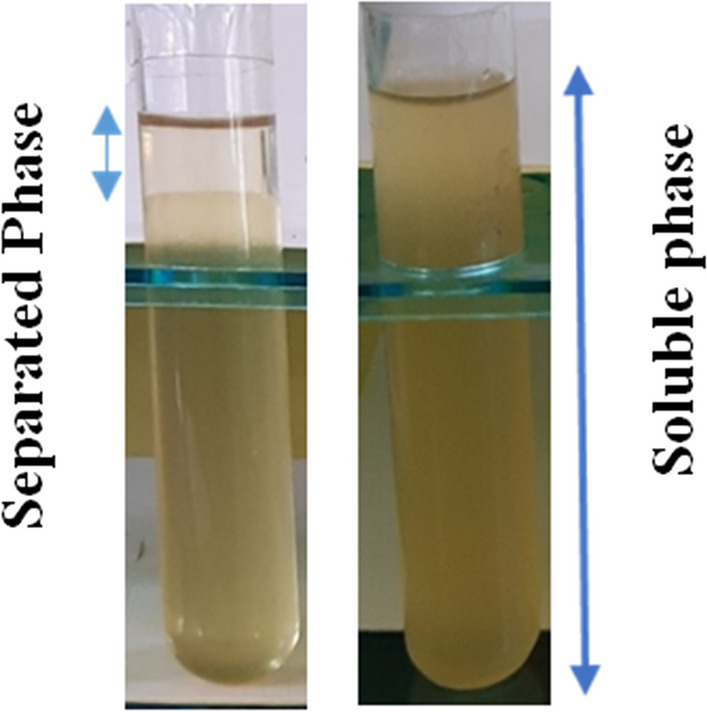
Stability of bioethanol in blend

After that, bioethanol stability in three blends was checked every 10 min for 1 h. The stability percentage of bioethanol in three blends is explained in Fig. 1 and was calculated according to the following equation:

$${\mathbf{S}}{\mkern 1mu} \left( \% \right) = \frac{{{\mathbf{Soluble}}{\mkern 1mu} {\mathbf{Phase}}{\mkern 1mu} - {\mkern 1mu} Separated{\mkern 1mu} Phase}}{{{\mathbf{Soluble}}{\mkern 1mu} {\mathbf{Phase}}}} \times 100$$

#### Image analysis and quantitative droplet analysis

To show fuel particles structure, all homogenized prepared samples were examined using a microscope (Leica Microsystems GmbH, Mannheim, Germany) [26]. No. of particles, average droplet size, and their distribution were measured and calculated using ImageJ software as [26]. First, the image can be opened using ImageJ software and analyzed by setting a scale (µm) on it. After that, the scale was cropped from the image, typed 8-bit, and adjusted by choosing Threshold. Then, particles were analyzed to determine No. of particles, average droplet size, and their distribution, Figs. (11, 12, 13, 14). Finally, droplet size distribution data was obtained from ImageJ software used for drawing histograms using Grapher software [27]. Some physical and chemical properties of produced bioethanol, biodiesel, and commercial diesel are listed in Table 1, as stated by [6].

**Table 1:** Produced bioethanol, biodiesel and commercial diesel properties [6, 20]

Properties/units	Produced bioethanol	Produced biodiesel	Commercial diesel	Norms
Viscosity/mm^2^s^−1^	1.24	3.52	2.39	ASTM D-445
Density (kg m^−3^)	0.8174	0.875	0.825	ASTM D-4052
Flashpoint (ºC)	8	4.03	55	ASTM D-93
Temperature of ignition (^o^C)	365	273	265	ASTM D-1929–20
Heating value/ MJkg^−1^	41.994	41.580	45.843	ASTM D-240
Point of cloud (ºC)	6	6	0	ASTM D-97
Cetane index	43	57	49	ASTM D-976

## Results and discussion

### Fuel blends stability

#### Visual investigation

**Fig. 2:**
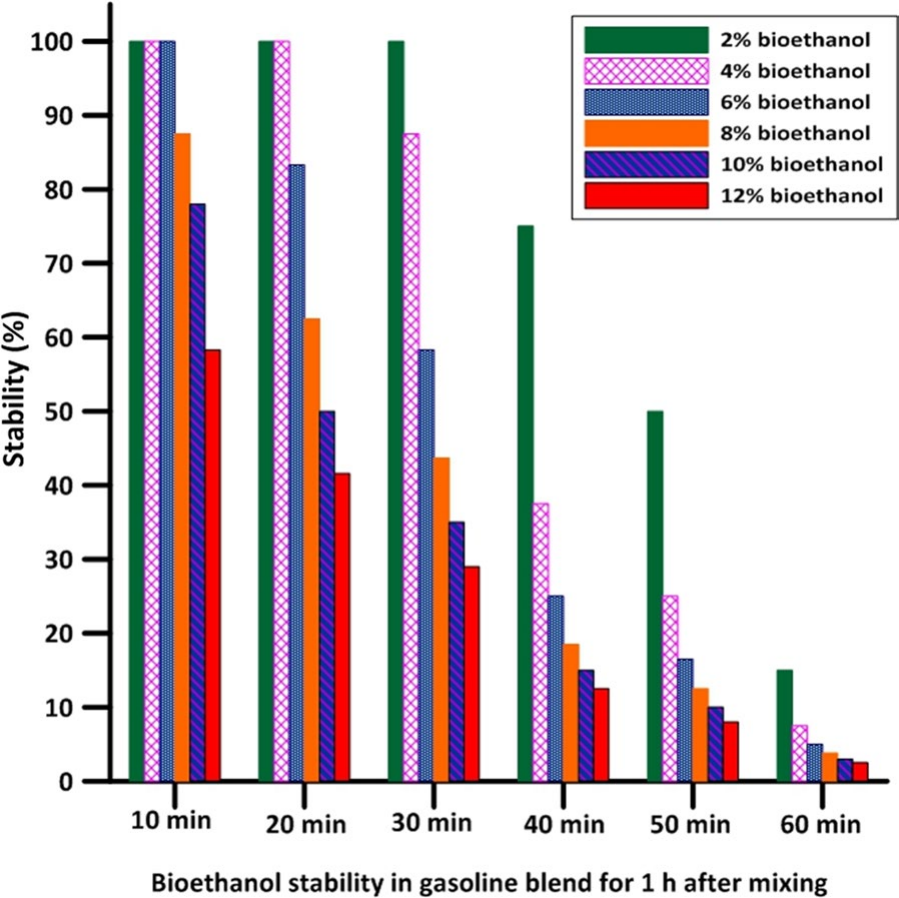
Stability percentage of bioethanol in gasoline blend for 1 h after mixing

**Fig. 3:**
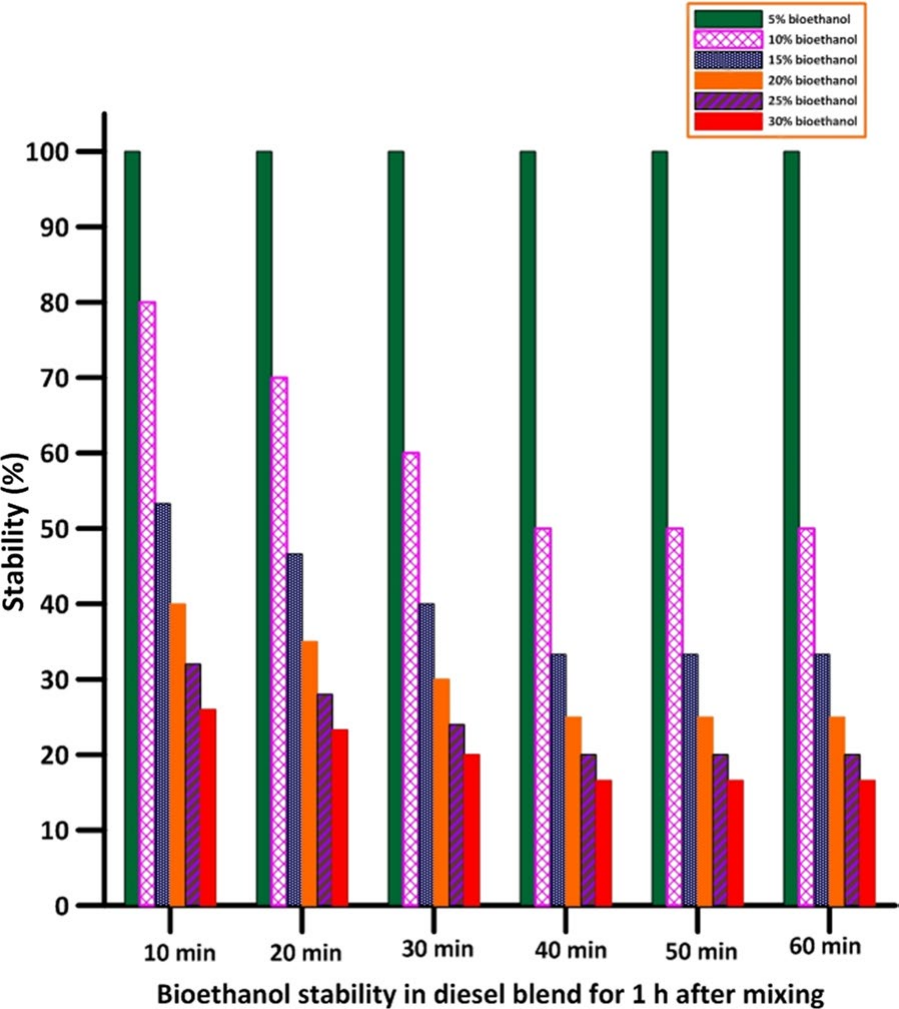
Stability percentage of bioethanol in diesel blend for 1 h after mixing

**Fig. 4:**
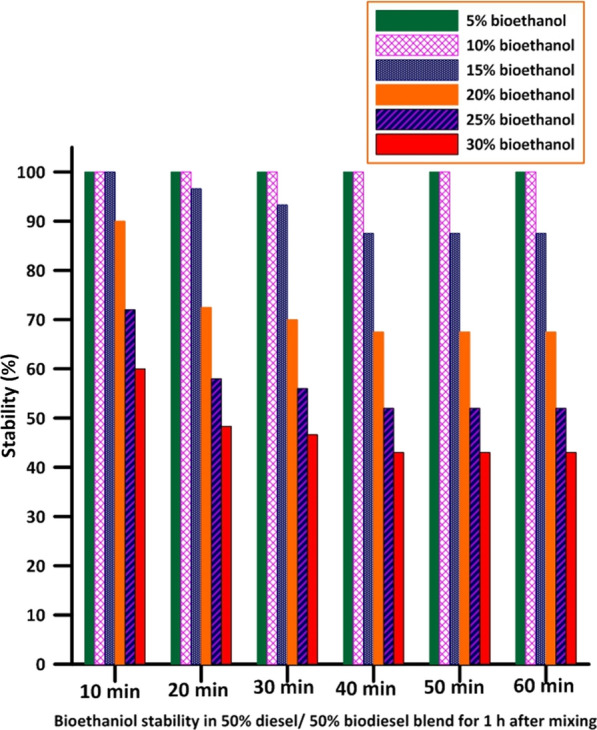
Stability percentage of bioethanol in 50% diesel/50% biodiesel blend or 1 h after mixing

**Fig. 5:**
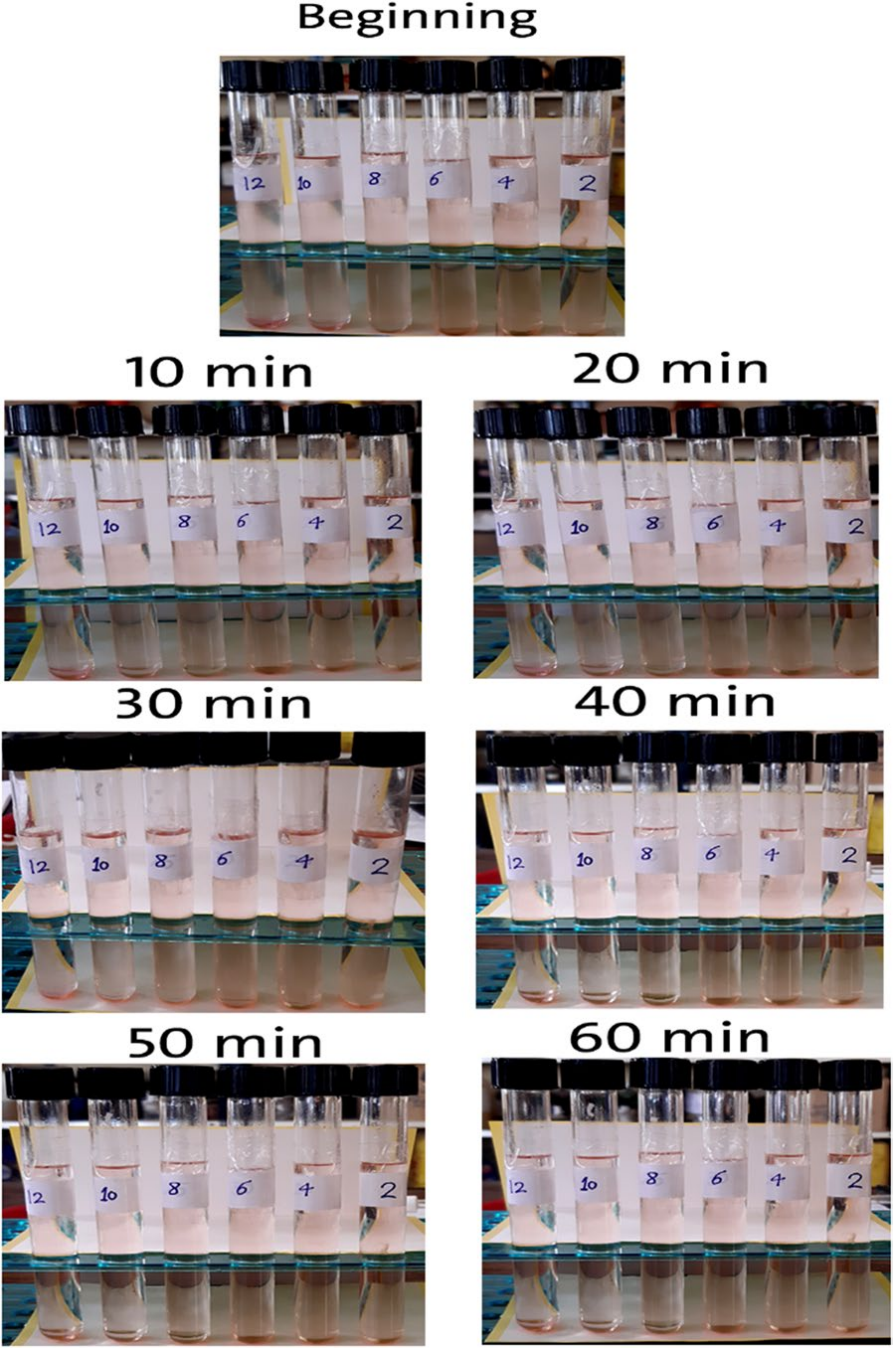
Bioethanol illustrative appearance and stability in gasoline blend for 1 h after mixing

**Fig. 6:**
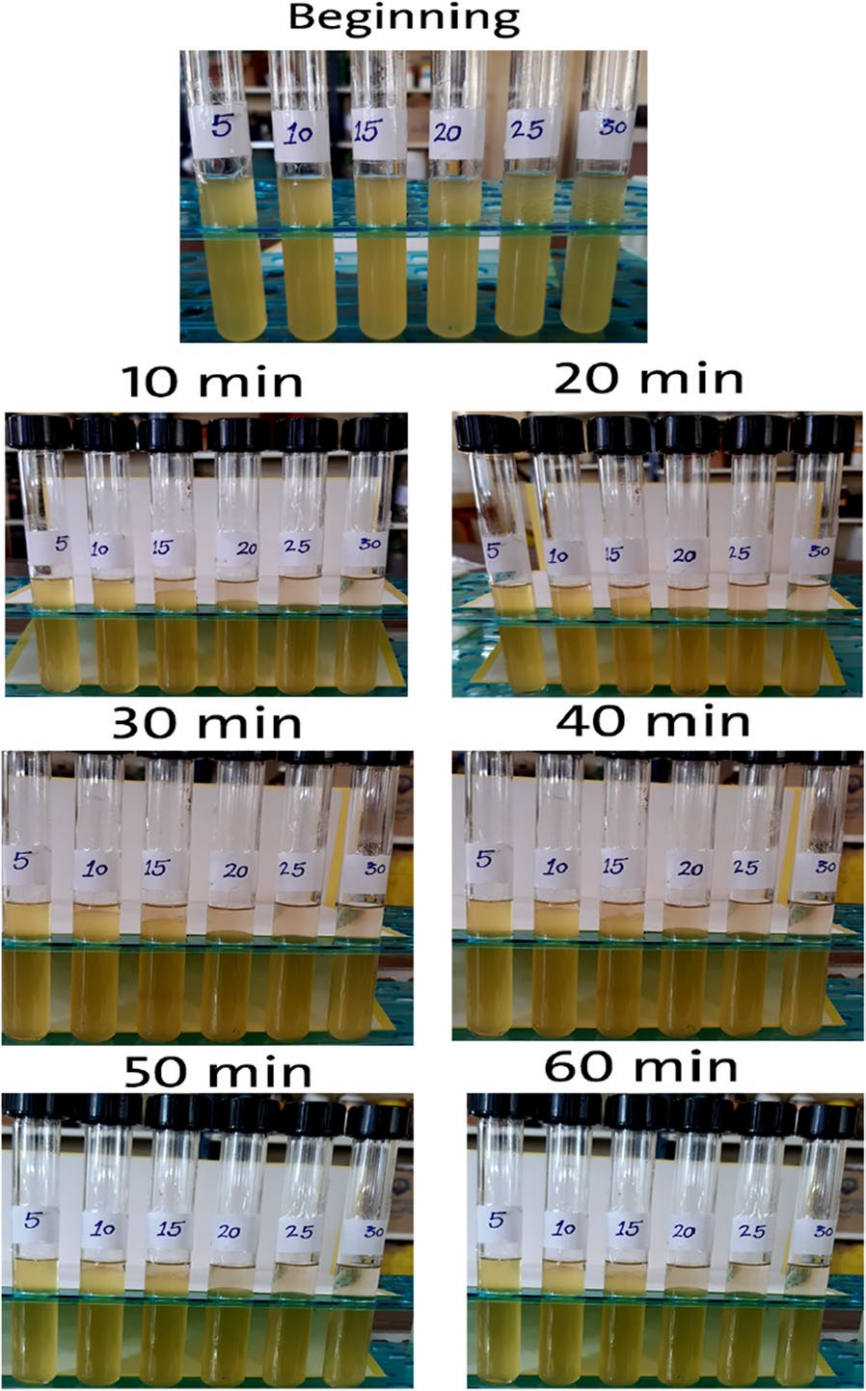
Bioethanol illustrative appearance and stability in diesel blend for 1 h after mixing

**Fig. 7:**
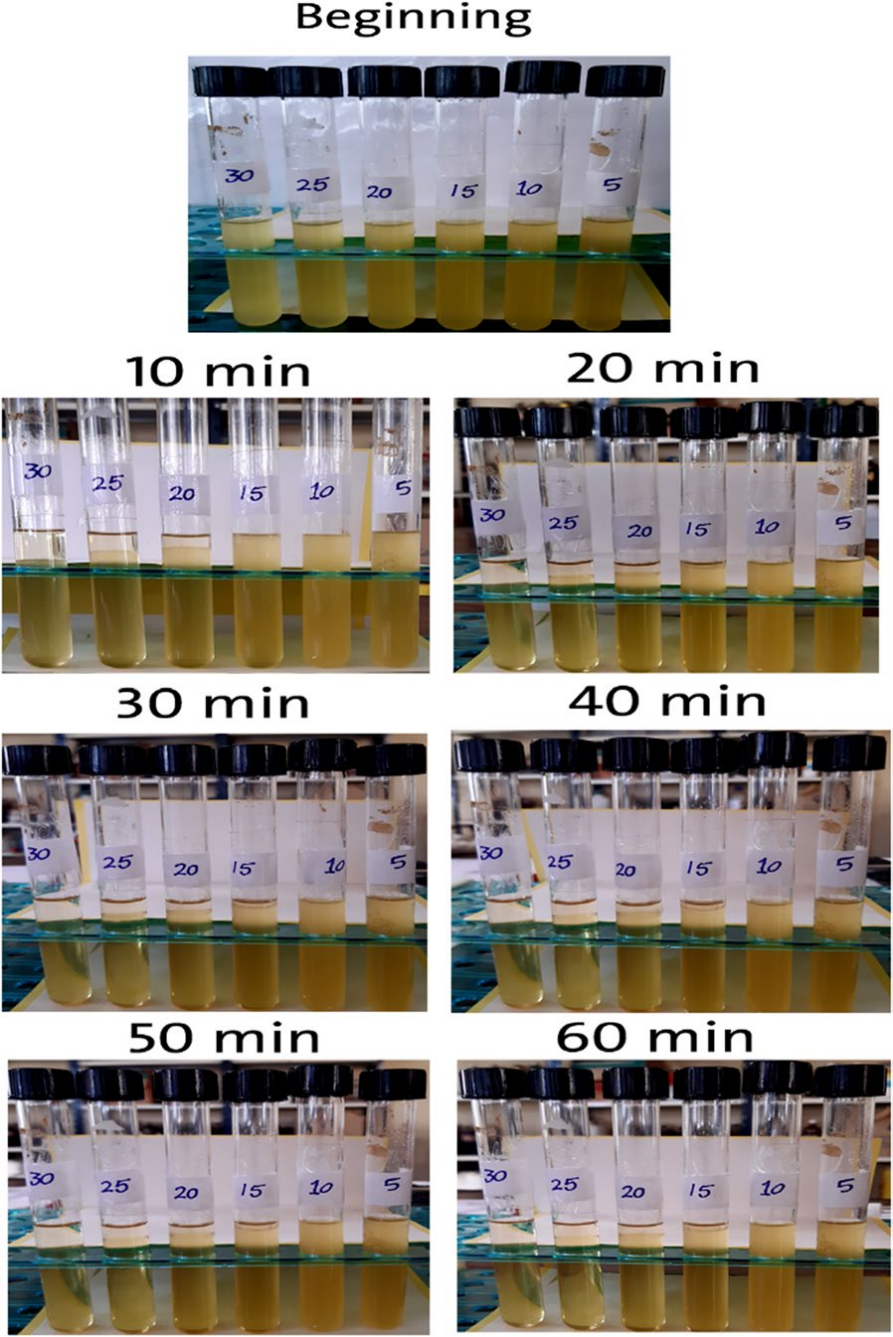
Bioethanol illustrative appearance and stability in (50%biodiesel/50%bioethanol) blend for 1 h after mixing

One of the main goals of applying fuel blends in engines is to keep engine modifications to a minimum, which necessitates blend stability and the formation of a single-phase liquid system [28]. Bioethanol stability in three different blends, “gasoline, diesel, and 50% biodiesel/50% bioethanol” is illustrated in Figs. 5, 6, 7 and Figs. 2, 3, 4. The obtained results showed that 50%biodiesel/50%bioethanol blend recorded the highest stability among other blends, and the lowest stability was recorded by the gasoline blend, followed by thediesel blend. After the first 10 min of mixing, concentrations of 8%, 10%, and 12% bioethanol by volume were separated from the gasoline blend, and the separation continued until complete separation was achieved after 1 h in all concentrations, as presented in Fig. 5. The obtained results revealed that the gasoline/bioethanol blends stability was very poor, although there are studies that used bioethanol blended with gasoline at a ratio of 10% and 20% [29]. This may have occurred under other conditions, such as adding additives to improve the blend’s stability or surfactants to lower the surface tension for ease of solubility or using an ultrasound-based mixing [30]. In our case, the prepared blend was separated into two layers after 10 min. For this reason, commercial diesel was selected to study its stability with prepared fuel.

In the diesel blend, all bioethanol concentrations were separated, and their stability in diesel was poor except for a concentration of 5%, which remained homogeneous with diesel even after 1 h of mixing, as shown in Fig. 6. In addition, results showed that the stability of diesel/bioethanol blends was better than the previous blend. However, because bioethanol and diesel fuels are not miscible, only a tiny amount of ethanol is allowed in the blending procedure for optimal results (less than 5%) [18], as shown in Fig. 5.

To overcome this problem, a surfactant or emulsifier should be employed to prevent bioethanol separation from diesel fuel and to enhance its amount [31]. Emulsifiers are materials that accumulate at the phase interface to reduce interfacial tension. Emulsifiers lower the energy needed to break dispersed phase in emulsion into droplets and stop them from collecting by creating a physical barrier or an attractive force between them [32]. Fatty Acid Methyl Esters (FAME) could also be employed as a surface-active compound to stabilize ethanol and Diesel mixtures [17]. Indeed, biodiesel prepared in the laboratory was blended with bioethanol and diesel, improving the stability as indicated by the previous studies. Concentrations (20%, 25%, and 30%) of bioethanol were separated from the 50%biodiesel/50%bioethanol blend, whereas the concentration of 15% showed a slight separation after 30 min, and the concentrations of 5% and 10% remained well homogenous with the blend (50% Diesel/50% biodiesel) even after 1 h, as shown in Fig. 7.

#### Droplet microstructure and phenomenological distribution

**Fig. 8:**
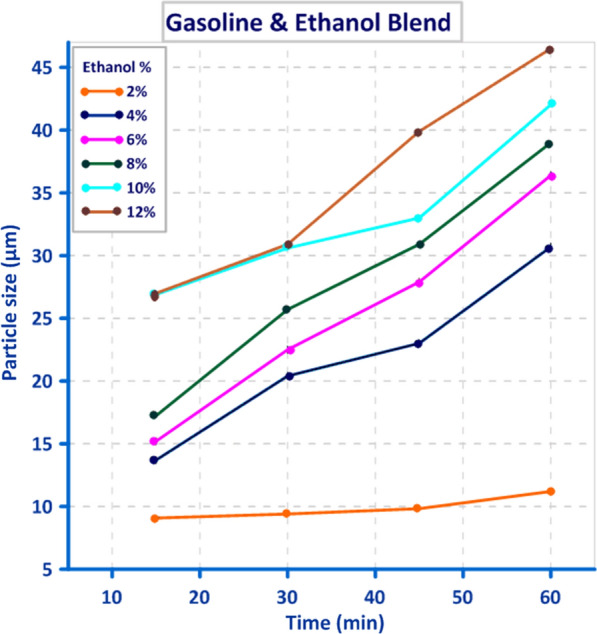
Effect of different bioethanol percentage to gasoline blend on particle size through 1 h

**Fig. 9:**
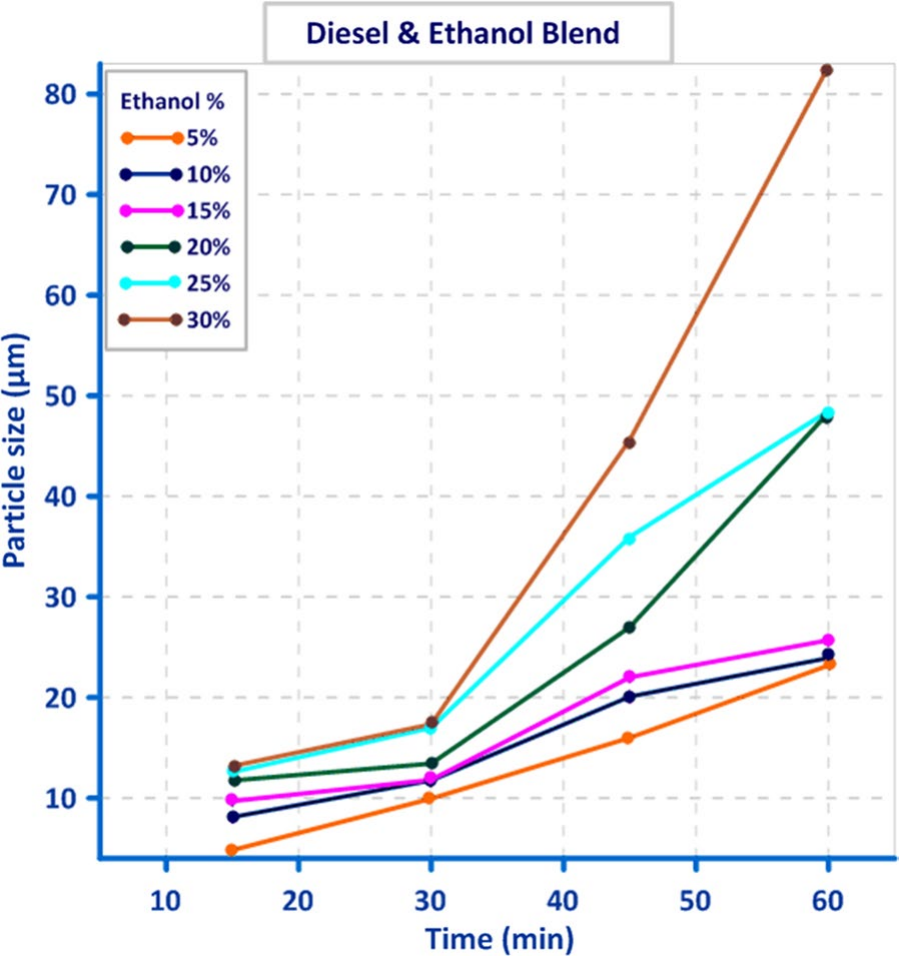
Effect of different bioethanol percentage to Diesel blend on particle size through 1 h

**Fig. 10:**
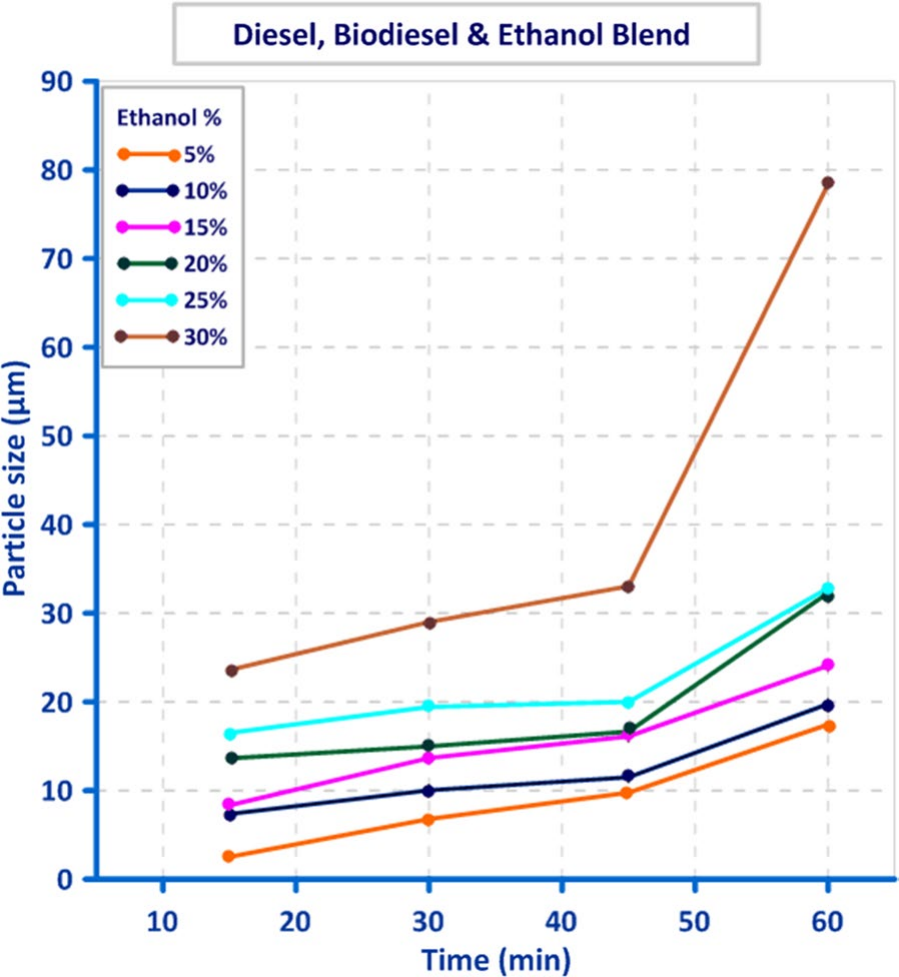
Effect of different bioethanol percentage to 50% diesel/50% biodiesel blend on particle size through 1 h

Droplet size distribution of three different blends, “gasoline, diesel and 50% biodiesel/50% bioethanol” is illustrated in Figs. 8, 9, and 10. The obtained result showed that there is a direct relation between Droplet size distribution and concentration of bioethanol inside the blend as time pass, this may be owing to the sudden expansion of particle size caused by the excess of bioethanol inside the blend [33]. From the perspective of the ethanol/water ratio as a hyperparameter for emulsion stability, this parameter can have a variety of effects, including the effects on surface activity indicated above as well as potential effects on the angle of contact between the polar phase and oil [26]. Previous studies suggested that while zein alone cannot stabilize oil-in-water emulsions, zein particles modified to be more hydrophilic do so [34]. In the gasoline blend, the results in Fig. 8 revealed that droplet size ranged from (9.1 µm) to (46.5 µm) using 2% bioethanol after 15 min and 12% bioethanol after 60 min, respectively. While the obtained results in Fig. 9 showed that utilizing 5% bioethanol after 15 min and 30% bioethanol after 60 min resulted in droplet sizes ranging from (4.8 µm) to (82.8 µm), respectively, in the Diesel blend. In 50% Diesel/50% Biodiesel blend, droplet size was ranged from (2.5 µm) to (78.4 µm) using 5% bioethanol after 15 min and 30% bioethanol after 60 min, respectively (Fig. 10).

In this study, when blending bioethanol with gasoline and diesel blends, their distribution was not uniform throughout the blend, and the maximum value of particle size was achieved. The particle size recorded the smallest value (2.5 µm) at 5% bioethanol after 15 min, and this value increased by increasing bioethanol concentration as time passed. In contrast, when bioethanol was mixed with a blend of 50% diesel and 50% biodiesel, particle distribution became uniform. This agrees with [14], who showed that the amount of ethanol could also alter how stable an emulsion is. Low ethanol concentrations can make emulsions more stable by reducing the size of the oil droplets as a result of reduced interfacial tension, whereas high ethanol concentrations alter the solubility of surfactants and can make an emulsion unstable [16]. In addition, [35] indicated that the region of stability of emulsions, in particular, was restricted to ethanol concentrations below 30%, where the emulsion displayed low viscosity and a monomodal droplet size distribution.

**Fig. 11:**
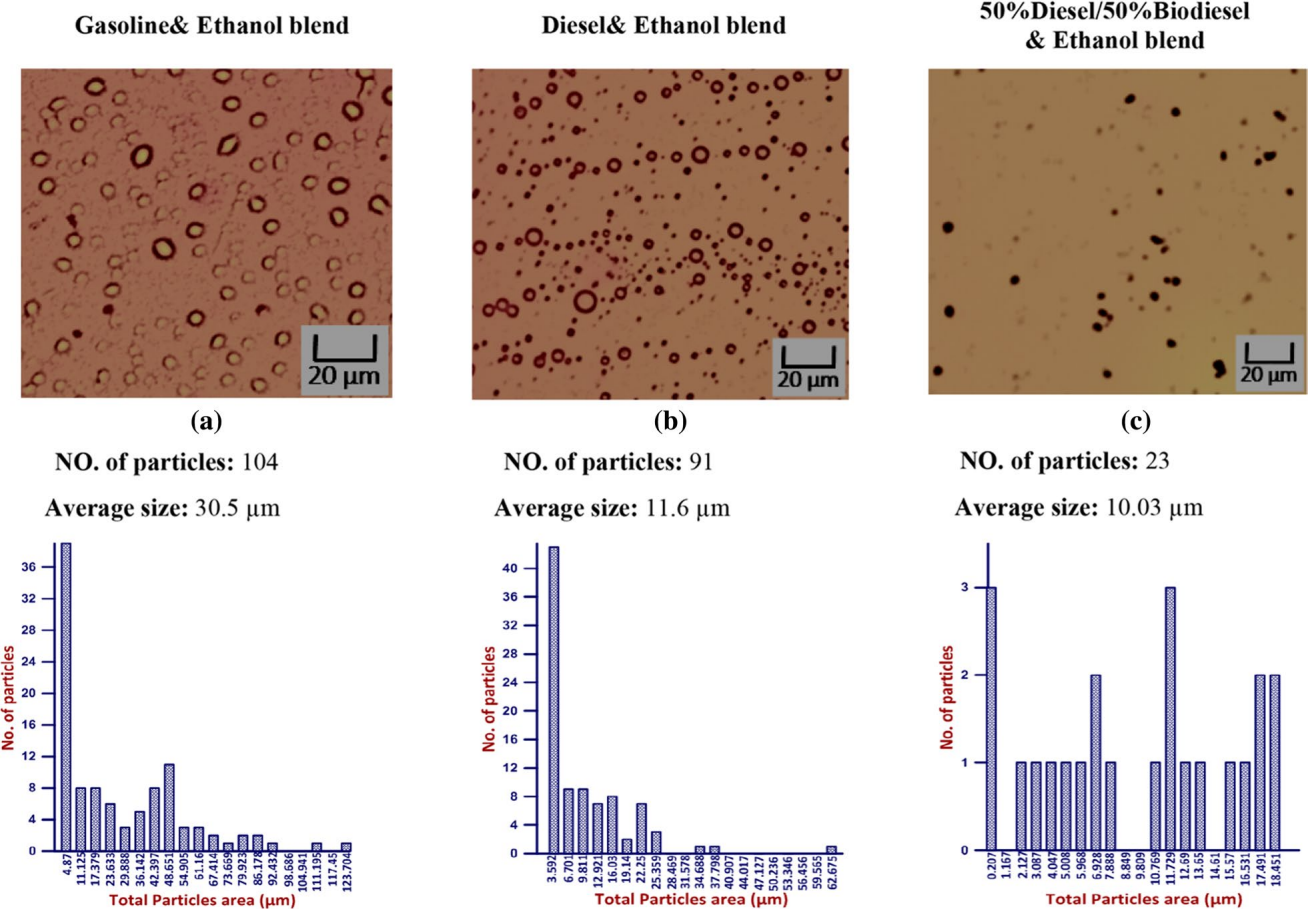
Effect of 10% Ethanol on gasoline, Diesel and (50%Diesel/50%Biodiesel) blends after 30 min

**Fig. 12:**
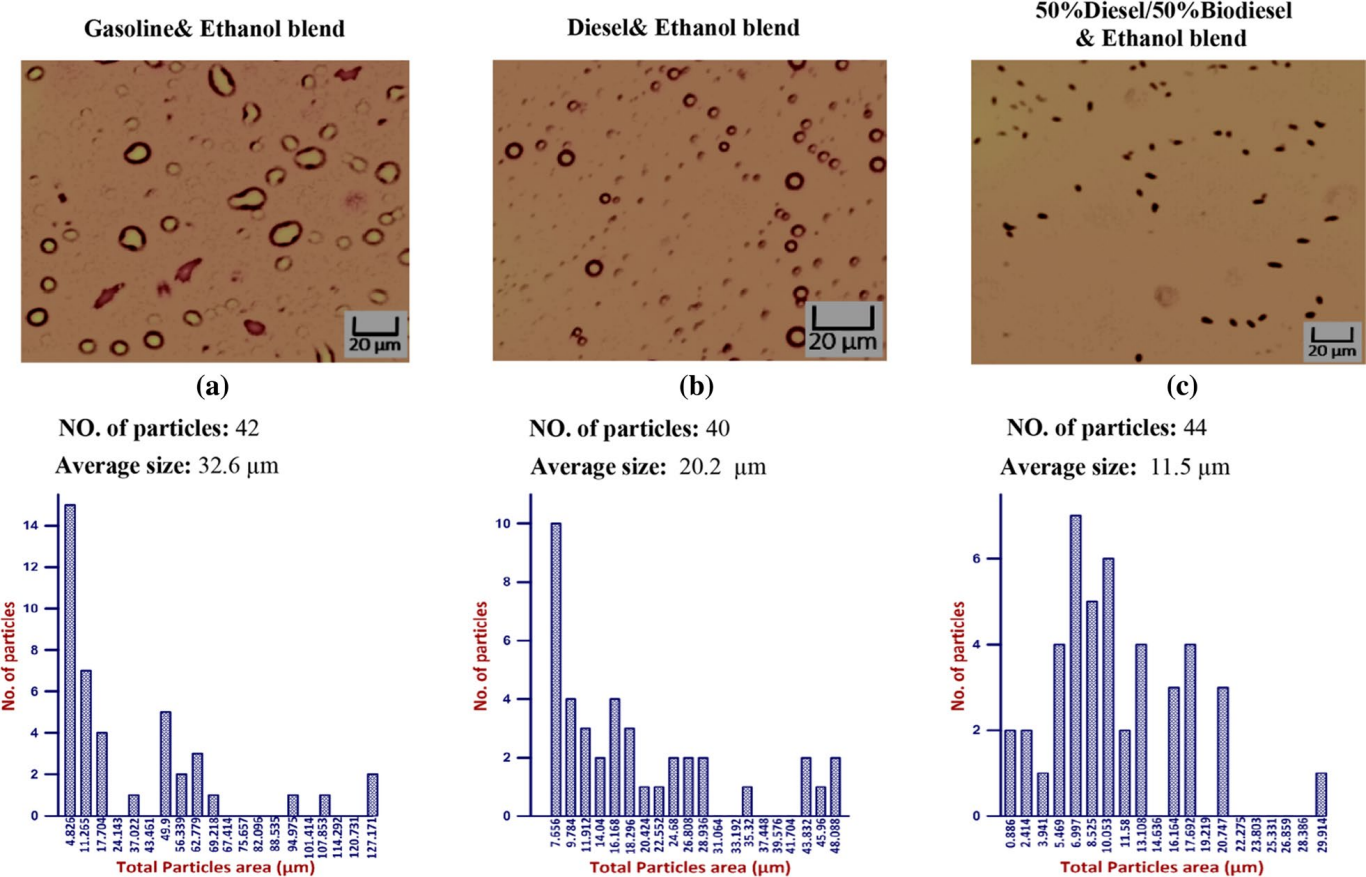
Effect of 10% Ethanol on gasoline, Diesel and (50%Diesel/50%Biodiesel) blends after 45 min

**Fig. 13:**
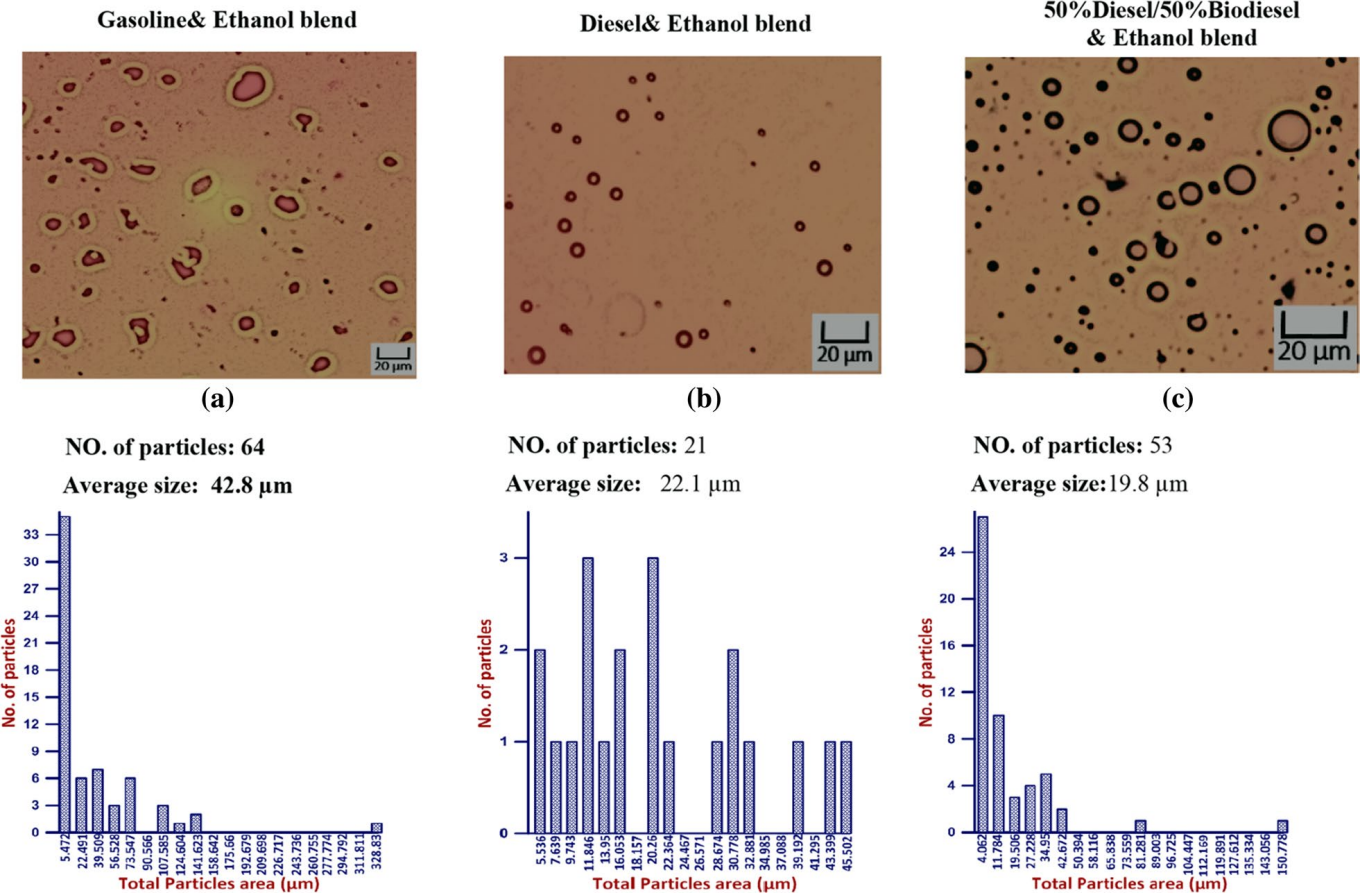
Effect of 10% Ethanol on gasoline, Diesel and (50%Diesel/50%Biodiesel) blends after 60 min

Ten % bioethanol for three different blends “Gasoline, Diesel and 50% Diesel/50% Biodiesel” after (30, 45 and 60 min) is illustrated in Figs. 11, 12, and 13 that contain pictures, no of particle, average size, and histogram for each measured sample. As time pass, the average size was increased inside each tested blend. Maximum average size was recorded using gasoline blend followed by Diesel and 50% Diesel/50% Biodiesel, as shown in Figs. 11, 12, and 13.

**Fig. 14:**
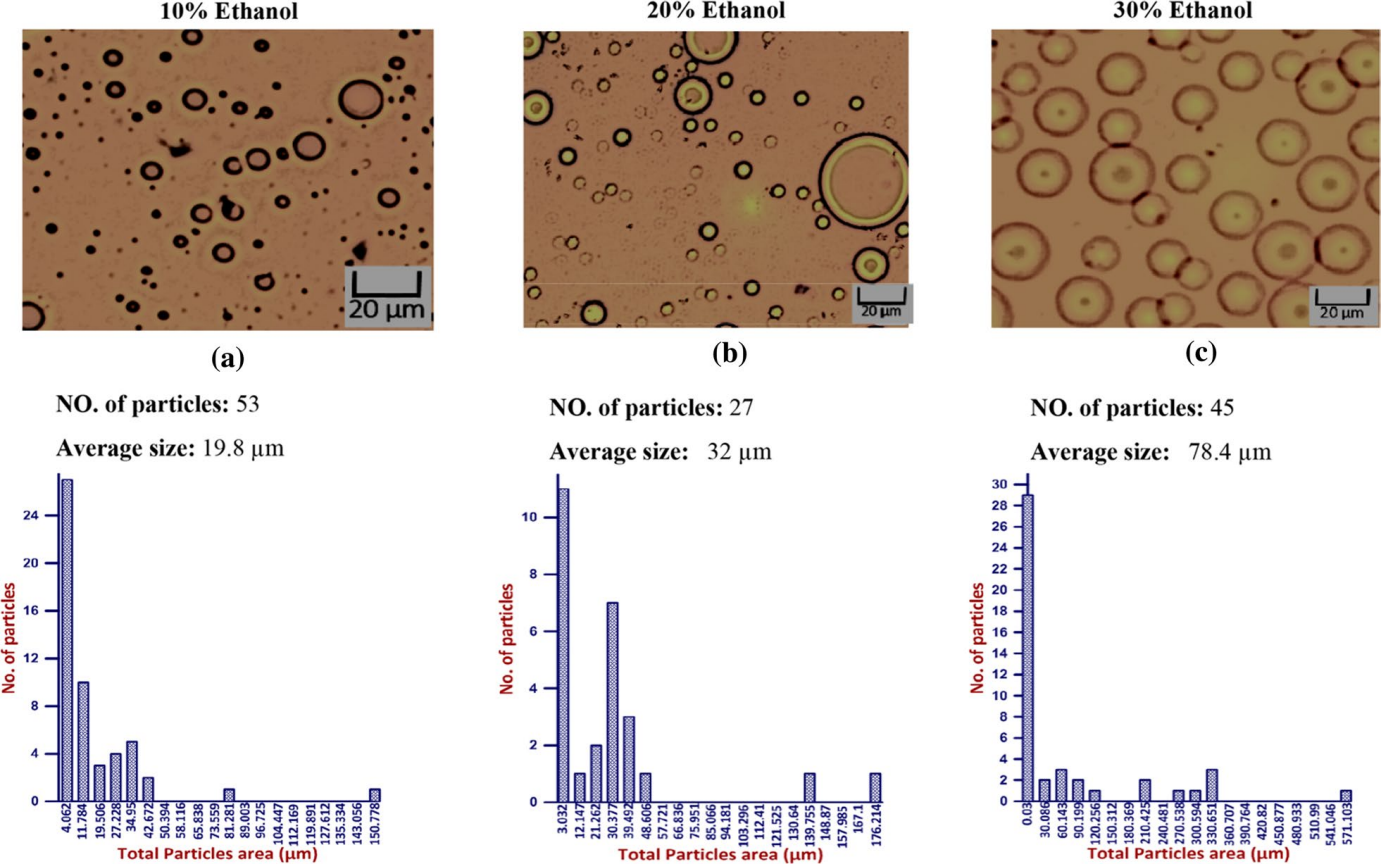
Effect of Ethanol blends to (50%Diesel/50percentageBiodiesel) blend on droplet size distribution

Results in Fig. 14 show that by increasing bioethanol concentration inside 50% Diesel/50% Biodiesel blend, the average size also increases. The average size for 10% bioethanol was 19.8 µm followed by 32 µm for 20% bioethanol and 78.4 µm for 30% bioethanol. Another study shows that the elevated ethanol dose percentage of 50–90% (based on permanent period of time) and enlarge in common droplet sizes are attend by an enlarge in the span of the droplet size distribution, where phase separation is limited [26]. However, creating the oil in the ethanol/water emulsions favors the supplementary oil drop infringement up further and becoming dispersed in the continuous water/ethanol phase. This is seen as a depression of phase inversion for emulsions produced by the gradual addition of oil compared to other studies’ direct emulsification of two volumes of oil and water [36]. As a result, the water/ethanol mixture will be in a continuous phase.

## Conclusions

This study revealed that the fuel particles stability and their distribution through blend were improved after using 50% diesel/50% biodiesel blend than pure diesel and gasoline. The most significant outcome of this research is that 5% and 10% bioethanol in 50% diesel/50% biodiesel blend were acceptable to be applied inside diesel engines. The size of bioethanol particles suspended through fuel blend, depending on the type of blend, the concentration of bioethanol mixed with the blend, and the time after mixing was detected by image analysis. The highest value of particle size was achieved when ethanol was mixed with gasoline and diesel blends, and their distribution was non-uniform throughout the blend. When bioethanol was mixed with 50% diesel/50% biodiesel blend, the distribution of particles became uniform, and particle size recorded the smallest value (2.5 µm) at 5% bioethanol after 15 min and this value increased by increasing bioethanol concentration as time pass.

## Data Availability

All data and materials are supplied in the manuscript.

## References

[1] El-Din H, Elkelawy M, Yu-Sheng Z (2010). HCCI engines combustion of CNG fuel with DME and H2 additives.

[2] Yu J, Yu-Sheng Z, Elkelawy M, Kui Q (2010). Spray and combustion characteristics of HCCI engine using DME/diesel blended fuel by port-injection.

[3] Elkelawy M, Alm-EldinBastawissi H, El Shenawy EA, Taha M, Panchal H, Sadasivuni KK (2021). Study of performance, combustion, and emissions parameters of DI-diesel engine fueled with algae biodiesel/diesel/n-pentane blends. Energy Convers Manag X.

[4] Becerra-Ruiz JD, Gonzalez-Huerta RG, Gracida J, Amaro-Reyes A, Macias-Bobadilla G (2019). Using green-hydrogen and bioethanol fuels in internal combustion engines to reduce emissions. Int J Hydrogen Energy.

[5] Amaral LV, Santos NDSA, Roso VR, Sebastião RdCdO, Pujatti FJP (2021). Effects of gasoline composition on engine performance, exhaust gases and operational costs. Renew Sustain Energy Rev.

[6] El-Sheekh MM, Bedaiwy MY, El-Nagar AA, ElKelawy M, Alm-EldinBastawissi H (2022). Ethanol biofuel production and characteristics optimization from wheat straw hydrolysate: performance and emission study of DI-diesel engine fueled with diesel/biodiesel/ethanol blends. Renew Energy.

[7] Han K, Lin Q, Liu M, Meng K, Ni Z, Liu Y (2022). Experimental study on the micro-explosion characteristics of biodiesel/1-pentanol and biodiesel/methanol blended droplets. Renew Energy.

[8] El-Seesy AI, El-Zoheiry RM, Fouad AK, Hussien AM, Elshabrawy SOM, He Z (2021). Impacts of octanol and decanol addition on the solubility of methanol/hydrous methanol/diesel/biodiesel/jet A-1 fuel ternary mixtures. RSC Adv.

[9] Amine M, Barakat Y (2021). Effect of cyclohexanol on phase stability and volatility behavior of hydrous ethanol-gasoline blends. Egypt J Petrol.

[10] Patni N, Pillai SG, Dwivedi AH (2013). Wheat as a promising substitute of corn for bioethanol production. Procedia Eng.

[11] Amine M, Awad EN, Ibrahim V, Barakat Y (2018). Influence of ethyl acetate addition on phase stability and fuel characteristics of hydrous ethanol-gasoline blends. Egypt J Petrol.

[12] Kyriakides A, Dimas V, Lymperopoulou E, Karonis D, Lois E (2013). Evaluation of gasoline–ethanol–water ternary mixtures used as a fuel for an otto engine. Fuel.

[13] Elsanusi OA, Roy MM, Sidhu MS (2017). Experimental investigation on a diesel engine fueled by diesel-biodiesel blends and their emulsions at various engine operating conditions. Appl Energy.

[14] Sun Y, Shen Y, Ding J, Ni X, Li C, Wang J (2022). High ethanol tolerance of oil-in-water pickering emulsions stabilized by protein nanoparticles. Colloids Surf A Physicochem Eng Aspects.

[15] Zeeb B, Herz E, McClements DJ, Weiss J (2014). Impact of alcohols on the formation and stability of protein-stabilized nanoemulsions. J Colloid and Interface Sci.

[16] Erxleben SWJ, Pelan E, Wolf B (2021). Effect of ethanol on the stability of sodium caseinate stabilised emulsions. Food Hydrocoll.

[17] Pradelle F, Leal Braga S, de Fonseca Aguiar Martins AR, Turkovics F, NohraChaarPradelle R (2019). Experimental assessment of some key physicochemical properties of diesel-biodiesel-ethanol (DBE) blends for use in compression ignition engines. Fuel.

[18] Mofijur M, Rasul MG, Hyde J, Azad AK, Mamat R, Bhuiya MMK (2016). Role of biofuel and their binary (diesel–biodiesel) and ternary (ethanol–biodiesel–diesel) blends on internal combustion engines emission reduction. Renew Sustain Energy Rev.

[19] El-Sheekh MM, Bedaiwy MY, El-Nagar AA, Elgammal EW (2022). Saccharification of pre-treated wheat straw via optimized enzymatic production using aspergillus niger: chemical analysis of lignocellulosic matrix. Biocatalysis Biotransformation.

[20] El Shenawy EA, Elkelawy M, Bastawissi HA-E, Panchal H, Shams MM (2019). Comparative study of the combustion, performance, and emission characteristics of a direct injection diesel engine with a partially premixed lean charge compression ignition diesel engines. Fuel.

[21] Elkelawy M, Alm-EldinBastawissi H, Esmaeil KK, Radwan AM, Panchal H, Sadasivuni KK (2019). Experimental studies on the biodiesel production parameters optimization of sunflower and soybean oil mixture and DI engine combustion, performance, and emission analysis fueled with diesel/biodiesel blends. Fuel.

[22] Elkelawy M, Etaiw SE-DH, Alm-EldinBastawissi H, Ayad MI, Radwan AM, Dawood MM (2021). Diesel/biodiesel/silver thiocyanate nanoparticles/hydrogen peroxide blends as new fuel for enhancement of performance, combustion, and emission characteristics of a diesel engine. Energy.

[23] Elkelawy M, Bastawissi H, Chandra Sekar S, Karuppasamy K, Vedaraman N, Sathiyamoorthy K, et al. Numerical and experimental investigation of ethyl alcohol as oxygenator on the combustion, performance, and emission characteristics of diesel/cotton seed oil blends in homogenous charge compression ignition engine. In: International Powertrains, Fuels & Lubricants Meeting. ISSN: 0148-7191, e-ISSN: 2688-3627, pp. 1–13. 10.4271/2018-01-1680.

[24] Gülüm M, Bilgin A (2018). A comprehensive study on measurement and prediction of viscosity of biodiesel-diesel-alcohol ternary blends. Energy.

[25] Gülüm M, Bilgin A (2021). Two-dimensional surface models to predict the density of biodiesel-diesel-alcohol ternary blends. Energy Sou Part Recov Util Environ Effects.

[26] Keshanidokht S, Via MA, Falco CY, Clausen MP, Risbo J (2022). Zein-stabilized emulsions by ethanol addition; stability and microstructure. Food Hydrocoll.

[27] Kamińska A, Roman M, Wróbel A, Gala-Błądzińska A, Małecki MT, Paluszkiewicz C (2022). Raman spectroscopy of urinary extracellular vesicles to stratify patients with chronic kidney disease in type 2 diabetes. Nanomed Nanotechnol Biol Med.

[28] Shahir SA, Masjuki HH, Kalam MA, Imran A, Fattah IMR, Sanjid A (2014). Feasibility of diesel–biodiesel–ethanol/bioethanol blend as existing CI engine fuel: an assessment of properties, material compatibility, safety and combustion. Renew Sust Energy Rev.

[29] El-Sheekha MM, Bedaiwy MY, El-ngar AA, Elgammal EW (2021). Isolation, identification and screening of cellulolytic activity of some fungi from different sources and localities in Egypt. Delta J Sci.

[30] Hoang AT, Tran VD (2019). Experimental analysis on the ultrasound-based mixing technique applied to ultra-low sulphur diesel and bio-oils. Int J Adv Sci Eng Inf Technol.

[31] Khalife E, Tabatabaei M, Demirbas A, Aghbashlo M (2017). Impacts of additives on performance and emission characteristics of diesel engines during steady state operation. Progress Energy Combust Sci.

[32] Ho TM, Razzaghi A, Ramachandran A, Mikkonen KS (2022). Emulsion characterization via microfluidic devices: a review on interfacial tension and stability to coalescence. Adv Colloid Int Sci.

[33] Yao C, Hu J, Geng P, Shi J, Zhang D, Ju Y (2017). Effects of injection pressure on ignition and combustion characteristics of diesel in a premixed methanol/air mixture atmosphere in a constant volume combustion chamber. Fuel.

[34] Feng Y, Lee Y (2016). Surface modification of zein colloidal particles with sodium caseinate to stabilize oil-in-water pickering emulsion. Food Hydrocoll.

[35] Espinosa GP, Scanlon MG (2013). Characterization of alcohol-containing dairy emulsions: pseudo-ternary phase diagrams of sodium caseinate solution-oil-ethanol systems. Food Res Int.

[36] Maffi JM, Meira GR, Estenoz DA (2021). Mechanisms and conditions that affect phase inversion processes: a review. Canadian J Chem Eng.

